# Down-regulation of UBC9 increases the sensitivity of hepatocellular carcinoma to doxorubicin

**DOI:** 10.18632/oncotarget.17939

**Published:** 2017-05-17

**Authors:** Sufen Fang, Junyao Qiu, Zheng Wu, Tao Bai, Wuhua Guo

**Affiliations:** ^1^ Department of Interventional Radiology, Mengchao Hepatobiliary Hospital of Fujian Medical University, Fuzhou 350025, China; ^2^ Department of Gastroenterology, The Second Affiliated Hospital of Nanchang University, Nanchang 330006, China

**Keywords:** UBC9, SUMOylation, hepatocellular carcinoma (HCC), doxorubicin (DOX), apoptosis

## Abstract

UBC9 is an E2-conjugating enzyme that is required for SUMOylation and has been implicated in regulating several critical cellular pathways. UBC9 is overexpressed in certain tumors, such as lung adenocarcinoma, ovarian carcinoma and melanoma, which implies that it has special clinical significance. However, the role of UBC9 in Hepatocellular carcinoma (HCC) drug responsiveness is not clear. In this study, we investigated the clinicopathological significance of UBC9 in HCC and investigated the mechanism of UBC9-mediated chemosensitivity to doxorubicin (DOX) in hepatocellular carcinoma cells. We found that relative to adjacent normal tissues, UBC9 was markedly overexpressed in HCC, which closely correlated with tumor size, tumor microsatellite formation, and tumor encapsulation. Our results also showed that down-regulation of UBC9 by shRNA reduced the expression of Bcl-2 and Bcl-xl and increased the expression of cleaved-Caspase3, which is a proapoptotic protein. These changes were associated with reduced apoptosis in response to DOX. Furthermore, we observed a mechanism involving modulation of the P38 and ERK1/2 signaling pathways. Together, our results indicate that down-regulation of UBC9 sensitizes cells to anticancer drugs, is possibly associated with the regulation of ERK1/2 and P38 activation and interacts with the intrinsic apoptosis pathway. Thus, knockdown of UBC9 may have a tumor suppressor effect and UBC9 could be a potential target for the treatment of HCC cancer.

## INTRODUCTION

Hepatocellular carcinoma (HCC) is the fifth most-common cancer and the second leading cause of cancer-related deaths worldwide [1]. However, in China in 2015, it was estimated that 111.5 thousand people died from HCC, which was the third leading cause of cancer-related deaths in China [2]. Due to its hidden onset, clinical diagnosis of hepatocellular carcinoma is more complex and there are few effective treatments. The effective treatments for HCC include surgery, intervention therapy and molecular target therapy, among a few other options [3]. Molecular target therapy is currently the primary focus of drug and treatment development for HCC [4], and this approach has led to breakthroughs in effective treatments for malignant tumors.”However, sorafenib, the sole medicine that targets HCC, can only increase the average survival rate of patients by approximately 3 months [4–6], and sorafenib has side effects, such as diarrhea, hand-foot skin disease, and high blood pressure [7, 8]. The poor efficacy and severe side effects seriously limit the use of sorafenib in clinical practice [9]. Therefore, new molecular target drugs with better efficacy and lower side effects should be studied further to develop additional treatments for HCC.

SUMO is a highly conserved protein family that is involved in protein modification after translation. Although SUMOylation is similar to ubiquitination in structure, conjugation process and attachment to target proteins, their biological consequences are different. Unlike ubiquitination, which normally targets protein degradation, SUMOylation has been implicated in the regulation of protein stability, protein–protein interactions, transcriptional activity and subcellular localization [10–11]. SUMOylation is a multi-step process that is catalyzed by multiple enzymes, including the E1, E2 and E3 enzymes [10–11]. In contrast to the ubiquitination pathway, which utilizes several E2 conjugating enzymes [12], UBC9 is the only known E2 conjugating enzyme in the SUMO pathway and has been found in yeast, invertebrates and vertebrates [13–15]. SUMOylation regulates a diverse array of cellular functions, including DNA replication and repair, chromosome integrity and segregation, kinetochore assembly, nuclear transport, signal transduction and cell cycle progression [16–21]. Many proto-oncogenic and tumor suppressor proteins are targets of SUMOylation, including Bcl2, PLAG1/PLAGL2, c-Fos, c-Myb and c-Jun, which plays a key role in regulating cancer cell proliferation and survival [20]. Some oncogenic signaling pathways were found to be regulated by SUMOylation, including MAPK, NF-κB, nuclear receptor transcription factors and their coregulators [22–24].

As the sole E2 enzyme, UBC9 is believed to play a central role in the biological processes of cell development through SUMOylation. UBC9 plays a crucial role in cell cycle regulation, apoptosis, DNA repair, gene transcription and nucleocytoplasmic transport [25, 26]. Other more recent studies have shown that UBC9 can function without depending on SUMOylation to regulate cell growth [27–28]. These studies suggested that regulating the expression of UBC9 would lead to changes in cell growth and function. In support of this notion, it was reported that UBC9 might regulate bcl-2 expression through the ER signaling pathway, which ultimately contributes to alterations in drug responsiveness and tumor growth [30]. It also has previously been shown that overexpression of UBC9-DN is associated with increased drug sensitivity in breast cancer [32]. In yeast, a defect in the ubc9 gene caused increased sensitivity to genotoxic drugs [33]. Therefore, the influence of the expression or function of UBC9 can impact drug responsiveness and tumorigenesis and alterations in protein SUMOylation and de-SUMOylation caused by UBC9 could affect cancer development and drug resistance.

Given the importance of UBC9 in tumor development and growth, we first demonstratedin this study that UBC9 was upregulated in HCC tissues compared to non-tumor tissues and showed that overexpression of UBC9 was associated with tumor aggressiveness and the grade of malignancy. We also studied whether down-regulated expression of UBC9 could increase the sensitivity of HCC cells to chemotherapy drugs. As shown in prior studies, doxorubicin (DOX) is widely used in HCC chemotherapy and mainly functions by preventing DNA replication and RNA synthesis, which ultimately inhibits tumor cell division and proliferation [34]. In this study, we aimed to determine if inhibition of UBC9 expression could increase the sensitivity of HCC cells to DOX and to examine the molecular mechanisms that were involved.

## RESULTS

### To explore the expression and significance of UBC9 in hepatic cancer (HCC) tissues

**Figure 1:**
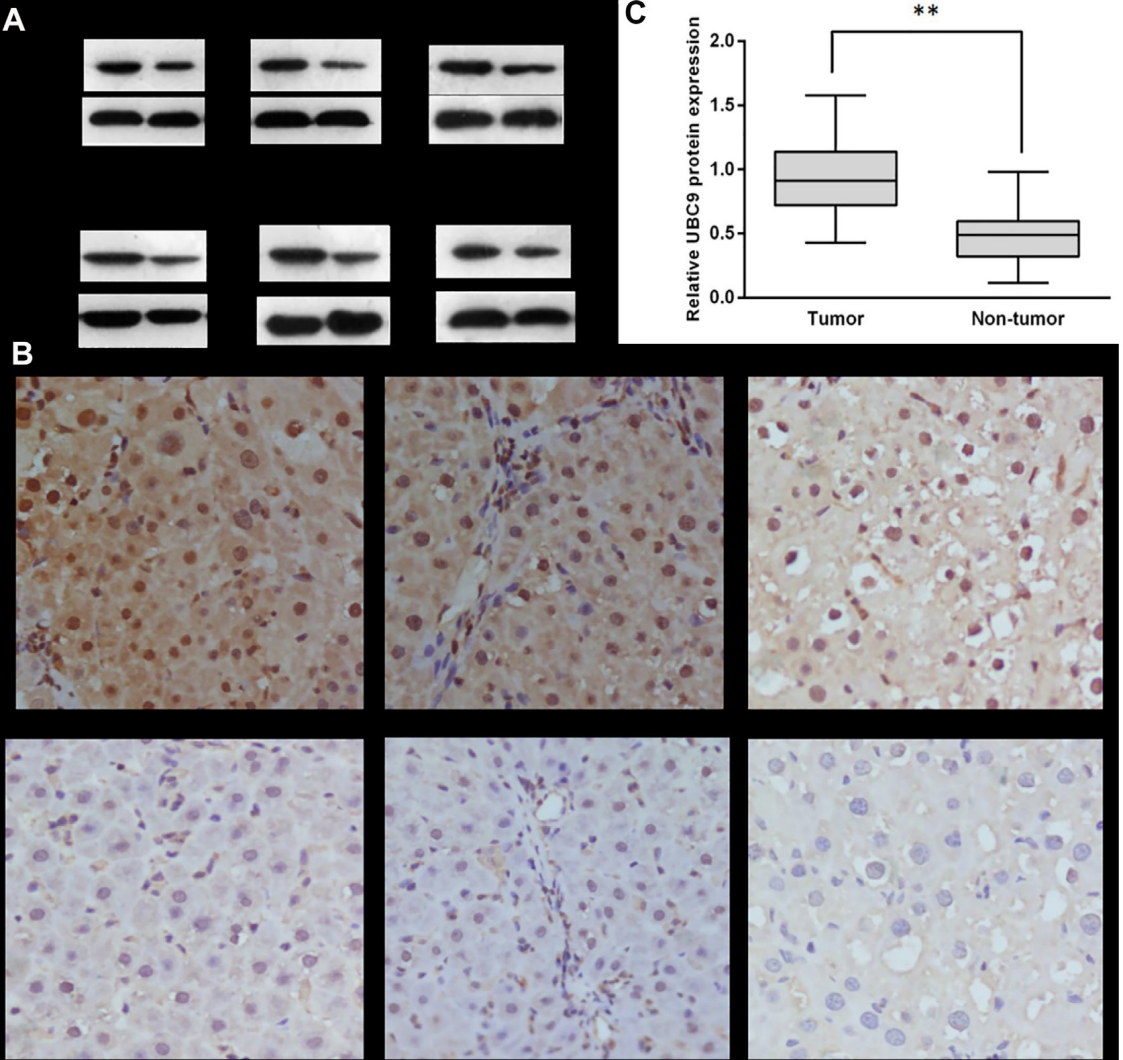
The expression of UBC9 in human HCC tumors and adjacent non-tumor liver tissues (**A**) Representative Western blot analysis of UBC9 protein expression (T: tumor, NT: non-tumor tissues). (**B**) Quantification of UBC9 protein expression using Western blot analyses for 103 paired HCC and their adjacent non-tumor tissues. GAPDH protein expression was used as an internal control (**P < 0.001). (**C**) Strong cytoplasmic and nuclear expression of UBC9 protein in 26th carcinoma tissues (a distant metastasis case); moderate cytoplasmic and nuclear expression of UBC9 protein in 56th carcinoma tissues (an intrahepatic metastasis case); weak cytoplasmic and nuclear expression of UBC9 protein in 85th carcinoma tissues (a case with small tumors and no transfer); almost no expression of UBC9 protein was seen in the adjacent non-tumor tissues.

The expression levels of UBC9 were determined for 103 patients with hepatic cancer by immunohistochemistry and Western blot. The results of immunohistochemistry and Western blot showed that the expression of UBC9 was significantly higher compared to that of the adjacent pericancerous liver tissues. The IHC results showed that the UBC9 protein was highly expressed in 69.92% (72/103) of the HCC tissue samples and in only 17.47 % (18/103) of the adjacent tissues (Figure 1C). Additionally, the western blotting results showed that the UBC9 protein levels were significantly elevated in HCC tissues (Figure 1A and 1B), which was consistent with the IHC results. These findings strongly indicate that UBC9 was overexpressed in HCC. The difference was statistically significant (*P* < 0.001). Next, we analyzed the association between UBC9 expression and the clinical pathological parameters in 103 HCC patients. The results showed that UBC9 overexpression correlated closely with tumor size, tumor microsatellite formation, and tumor encapsulation (*P* < 0.05 for all; Table 1). These results indicated that UBC9 overexpression was involved in HCC aggressiveness and the grade of malignancy. Additional experiments were needed to determine whether the status of UBC9 overexpression might be an independent factor.

**Table 1:** The *P* values represent probabilities for UBC9 expression levels between variable subgroups determined by the χ2 test

Pathologic Characteristics	*N*	UBC9		*P* value
		Overexpression (number of cases)	Nonoverexpression (number of cases)	
**Age, years**				
≤ 60	69	46	23	0.095
> 60	34	26	8	
Sex				
Male	86	68	18	0.239
Female	17	8	9	
**Tumor size (cm)**				
≤ 5	45	26	19	0.001
> 5	58	46	12	
**Tumor microsatellite formation**				
Absent	66	37	29	< 0.001
Present	37	35	2	
**HBsAg**				
Negative	13	11	2	0.216
Positive	90	61	29	
**AFP (ng/ml)**				
≤ 400	51	36	15	0.914
> 400	52	36	16	
**Cirrhosis**				
Absent	39	26	13	0.576
Present	64	46	18	
**Tumor encapsulation**				
Absent	15	5	10	0.001
Present	88	67	21	
**Portal vein tumor thrombosis**				
Absent	93	64	29	0.546
Present	10	8	2	

Abbreviations: AFP, a-fetoprotein; HBsAg, hepatitis B surface antigen.

### Analysis of UBC9 expression with UBC9 shRNA in HCC cells

**Figure 2:**
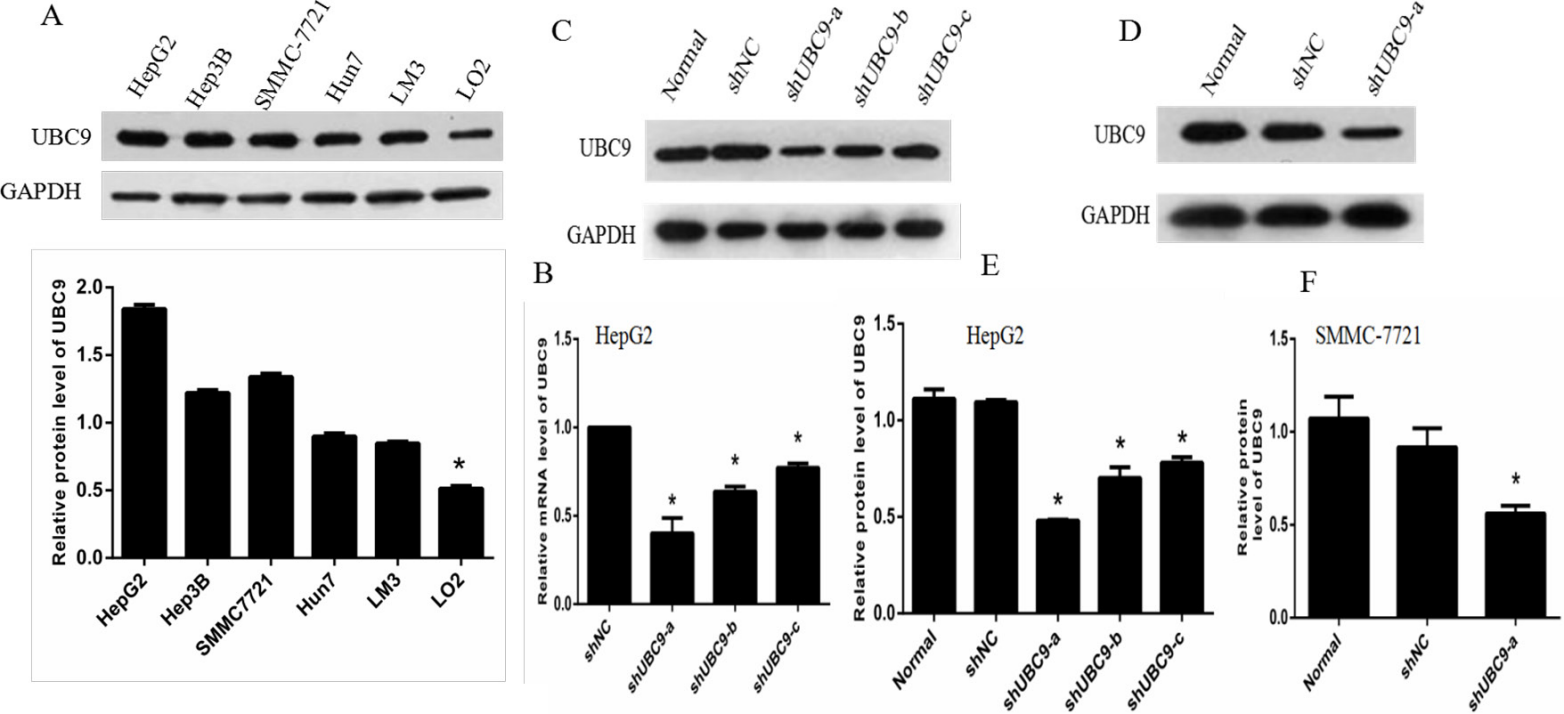
UBC9 expression in untreated and treated groups of HCC cells (**A**) Western blot analyses of UBC9 expression in the indicated HCC cell lines and the normal liver cell line LO2.*P < 0.001vs. Each hepatoma cell lines. (**B**) The mRNA expressions of UBC9 in HepG2 cells with UBC9-shRNA were determined by qRT-PCR.*P < 0.01 vs NC shRNA transected cells. (**C** and **D**) Protein expression of UBC9 was examined using Western blots in HepG2 and SMMC-7721 cells. *P < 0.001 vs NC shRNA transected cells. (**E** and **F**) Graph of the relative ratios of UBC9 protein to GAPDH in each group. *P < 0.001vs. NC shRNA transected cells.

We first examined the levels of UBC9 in a variety of HCC cells by Western blotting. The results indicated that the expression of UBC9 in HCC cells was higher than that in normal liver cells (Figure 2A). Then, we stably transfected a UBC9-specific short hairpin RNA (shUBC9) into in HepG2 and SMMC-7721 cells, which exhibit relatively high expression of UBC9 among HCC cell lines (Figure 2A). Western blotting and qRT-PCR showed that the UBC9 levels significantly declined in HepG2 cells (Figure 2B–2D). The amounts of UBC9 mRNA and protein were significantly reduced in HepG2 cells transfected with one UBC9 shRNA (shUBC9-a), which showed that efficient knockdown of UBC9 occurred. Similar results were observed in cells transfected with another UBC9 shRNA (shUBC9-b), although the effect of UBC9 downregulation was smaller. However, the UBC9 expression levels were only slightly affected by the transfection of UBC9 shRNA (shUBC9-c). The amounts of UBC9 protein were significantly reduced in SMMC-7721 cells transfected with one UBC9 shRNA (shUBC9-a) (Figure 2E and 2F), These results suggested that UBC9 shRNA could substantially reduce UBC9 expressions in HCC cells though transfection of UBC9-a shRNA.

### The influence on proliferation after transfection of UBC9 shRNA in combination with doxorubicin treatment in HCC cells

**Figure 3:**
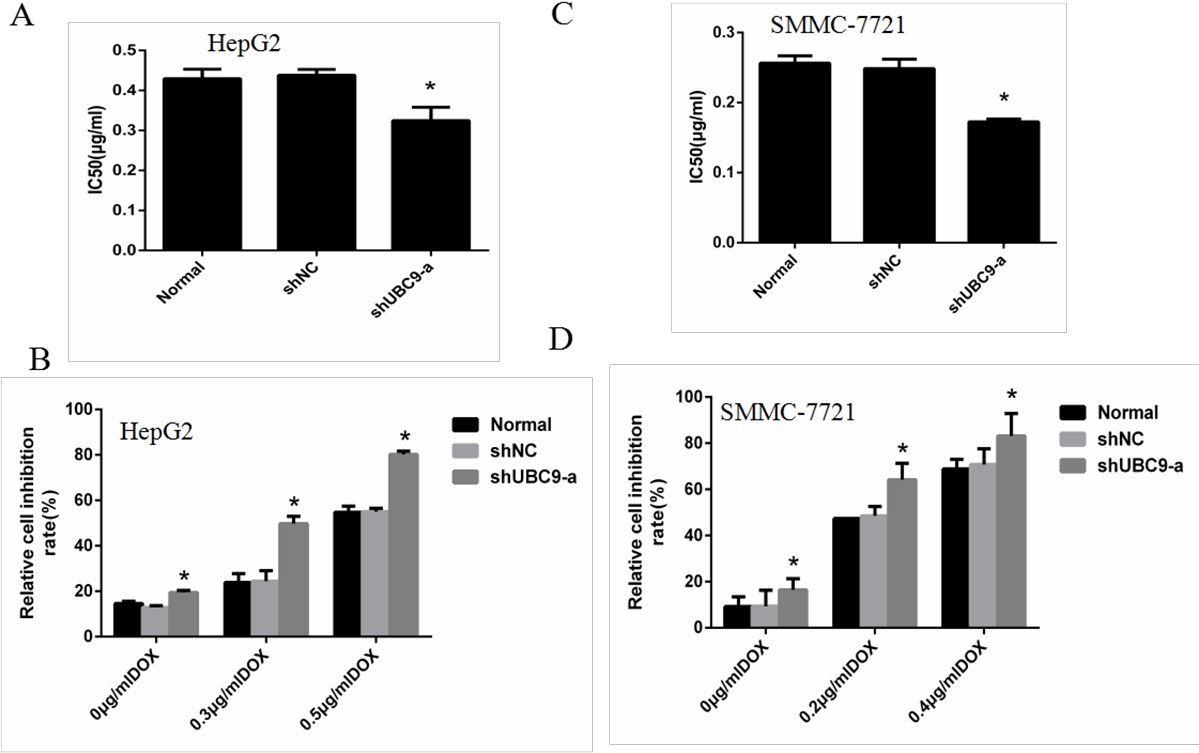
Down-regulation of UBC9 sensitized HCC cells to Doxorubicin (**A** and **C**) The IC50 values of HepG2 and SMMC-7721 cellswere determined according to the plot.*p < 0.01 vs NC shRNA transfected cells, *n* = 3. (**B** and **D**) The viability was measured by CCK8. The cell viability experiments revealed that treatment of HepG2 and SMMC-7721 cells with UBC9-shRNA resulted in more enhanced sensitivity to doxorubicin in comparison to Normal cells and NC shRNA transfected cells. The values are mean ± standard deviation. *P < 0.001 vs NC shRNA transfected cells.

To address the role of UBC9 in chemosensitivity of HCC cells, HCC cells were transfected with UBC9-shRNA or NC-shRNA. The G418-resistant mix clones were selected for further experiments. Then the cells were treated with different concentrations of DOX (0,0 −1.6 μg for 24 h. The cell viability in the presence of DOX was further evaluated with CCK8 assay. It was indicated down-regulation of UBC9 resulted in poor cell viability (Figure 3B). The IC50 values of HCC cells to DOX were calculated from the cell viability plots. For hepG2 cells, IC50 values of shUBC9-a group cells were decreased compared to NC group (*P* < 0.05); but there was no significant difference between the Normal and shNC group cells (Figure 3A), For SMMC-7721, As displayed by Figure 3C, suppression of UBC9 was also accompanied by significantly decreased IC50 values. These data all indicated that down-regulation of UBC9 could increase sensitivity of HCC cells to DOX.

To study the inhibition rate of DOX in HCC cells, we used the three groups of HepG2 and SMMC7721 cells respectively transfected with 0, 0.3, 0.6 and 0, 0.2, 0.4 μg/ml DOX for 24 h to assess the cell viability by CCK8 assays. The shUBC9-a group combined with doxorubicin treatment had a significantly increased inhibition rate (*P* < 0.01) compared to shNC group cells treated with doxorubicin. (Figure 3B, 3D)

### The influence of the apoptosis associated protein expression after UBC9 shRNA was combined with DOX treatment in HCC cells

**Figure 4:**
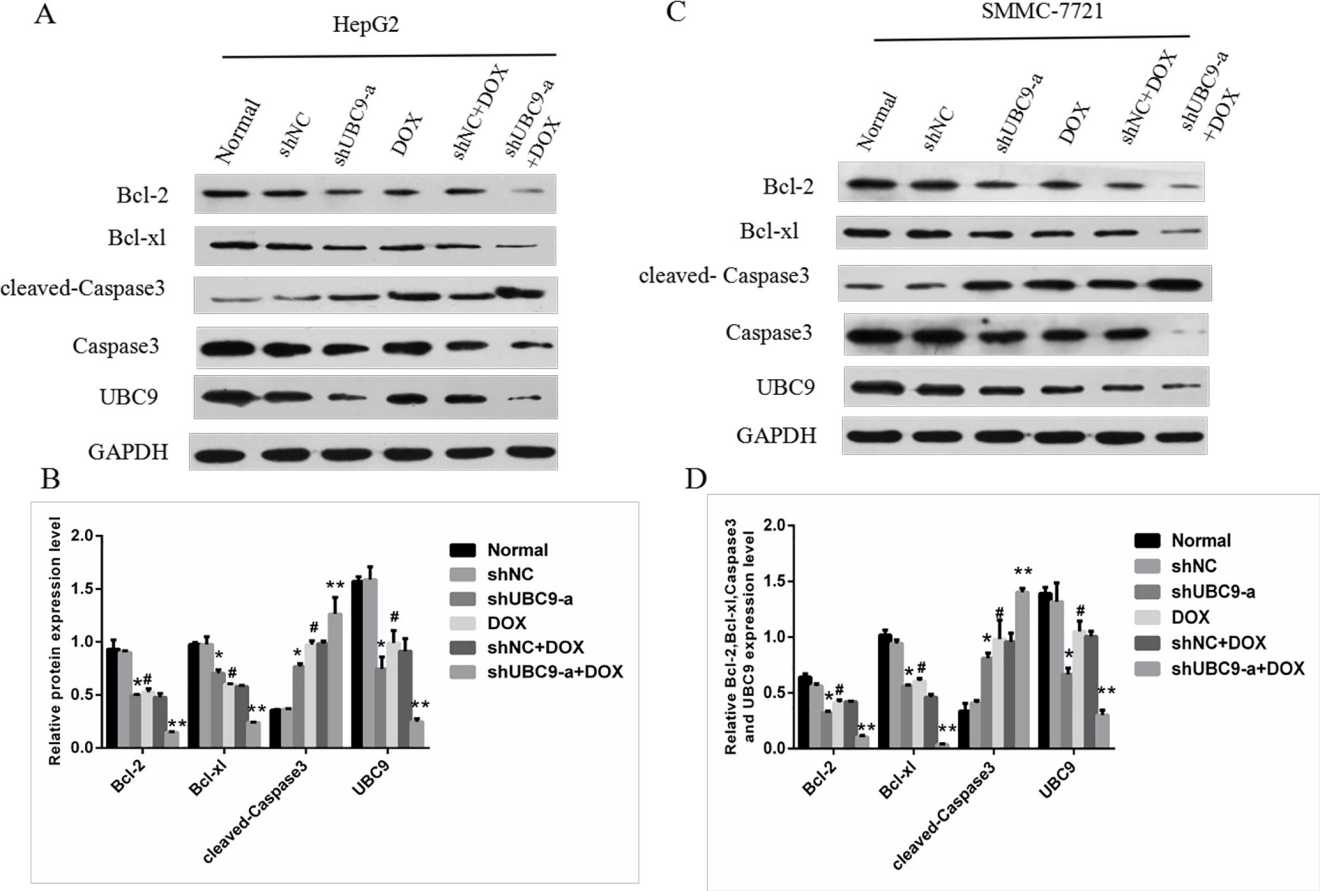
The effect of UBC9 on apoptosis-related proteins in HCC cells (**A** and **C**) The expression of cleaved-Caspase3, Caspase3,Bcl-2, UBC9 and Bcl-xl in HepG2 and SMMC-7721 cells were evaluated by Western blot. (**B** and **D**) Graph of the relative ratios of cleaved-Caspase3,Bcl-2, UBC9 and Bcl-xl protein to GAPDH in each group. *P < 0.05 compared to shNC cells without DOX, **P < 0.001 compared to shNC cells with DOX .^#^*P* < 0.001 vs Normal cells without DOX, *n* = 6.

we manipulated the apoptosis associated protein levels by the stable transfection of UBC9shRNA or vector control into HepG2 and SMMC-7721 cells. Western blotting showed that the knockdown of UBC9 reduced the expression of Bcl-2 and Bcl-xl and increased the expression of cleaved-Caspase3 in HepG2 and SMMC-7721 cells (Figure 4A–4D). The Ct values of the Bcl-2, Bcl-xl and UBC9 protein were significantly decreased in the shUBC9-a and shUBC9-a with DOX groups (*P* < 0.001), and the shUBC9-a with DOX group had a greater reduction compared to shUBC9 (*P* < 0.001). The Ct values of the cleaved-Caspase3 protein were significantly increased in the shUBC9-a and shUBC9-a with DOX groups (*P* < 0.001), and the shUBC9-a with DOX group had a greater increased compared to the shUBC9 group (*P* < 0.001).

### The influence of apoptosis after UBC9 shRNA in combination with DOX treatment in HCC cells

**Figure 5:**
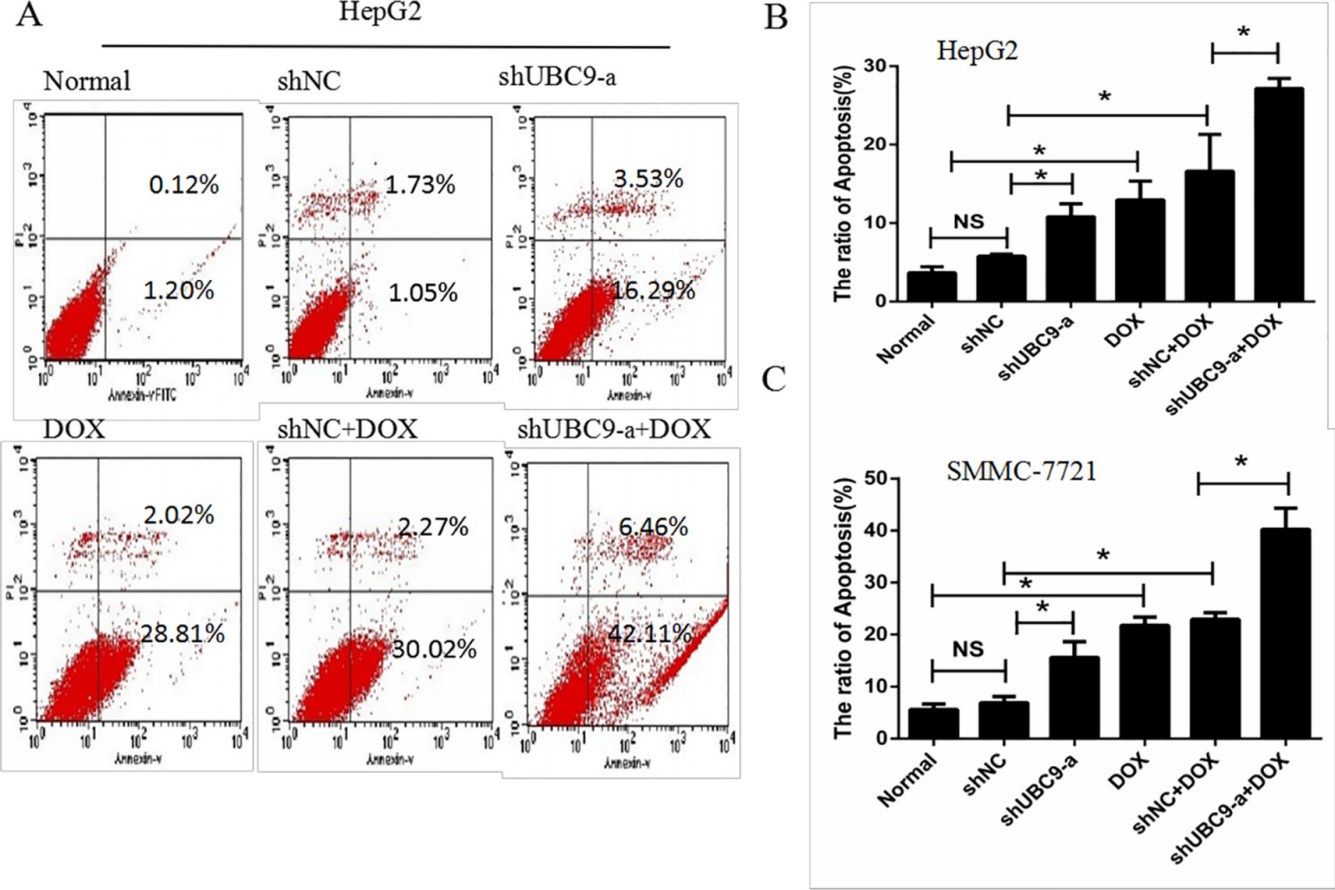
Down-regulation of UBC9 enhanced DOX-induced apoptosis in HCC cells (**A**) Cell apoptosis was analyzed with flow cytometry, and the percentage of apoptotic cells was determined according to cells expressing Annexin. (**B** and **C)** Early and late apoptotic cells were increased in UBC9 shRNA in combination with DOX compared to other group cells. *P < 0.001 vs NC shRNA transfected cells *n* = 6.

We investigated whether the effects of shUBC9-a with DOX treatment increased apoptosis using the Annexin V-FITC/PI staining method. We used three groups of cells alone or treated with doxorubicin for 24 h. UBC9 knockdown or DOX treatment led to a marked increase in cell apoptosis; however, combination of UBC9 knockdown with DOX induced a higher cell apoptosis than chemotherapy group alone in HepG2 and SMMC7721 (Figure 5A–5C). These findings suggested that down-regulation of UBC9 expression increased the chemosensitivity of doxorubicin-treated HCC cells by inducing apoptosis.

### The effects on cell cycle progression after UBC9 shRNA combined with DOX Treatment in HCC cells

**Figure 6:**
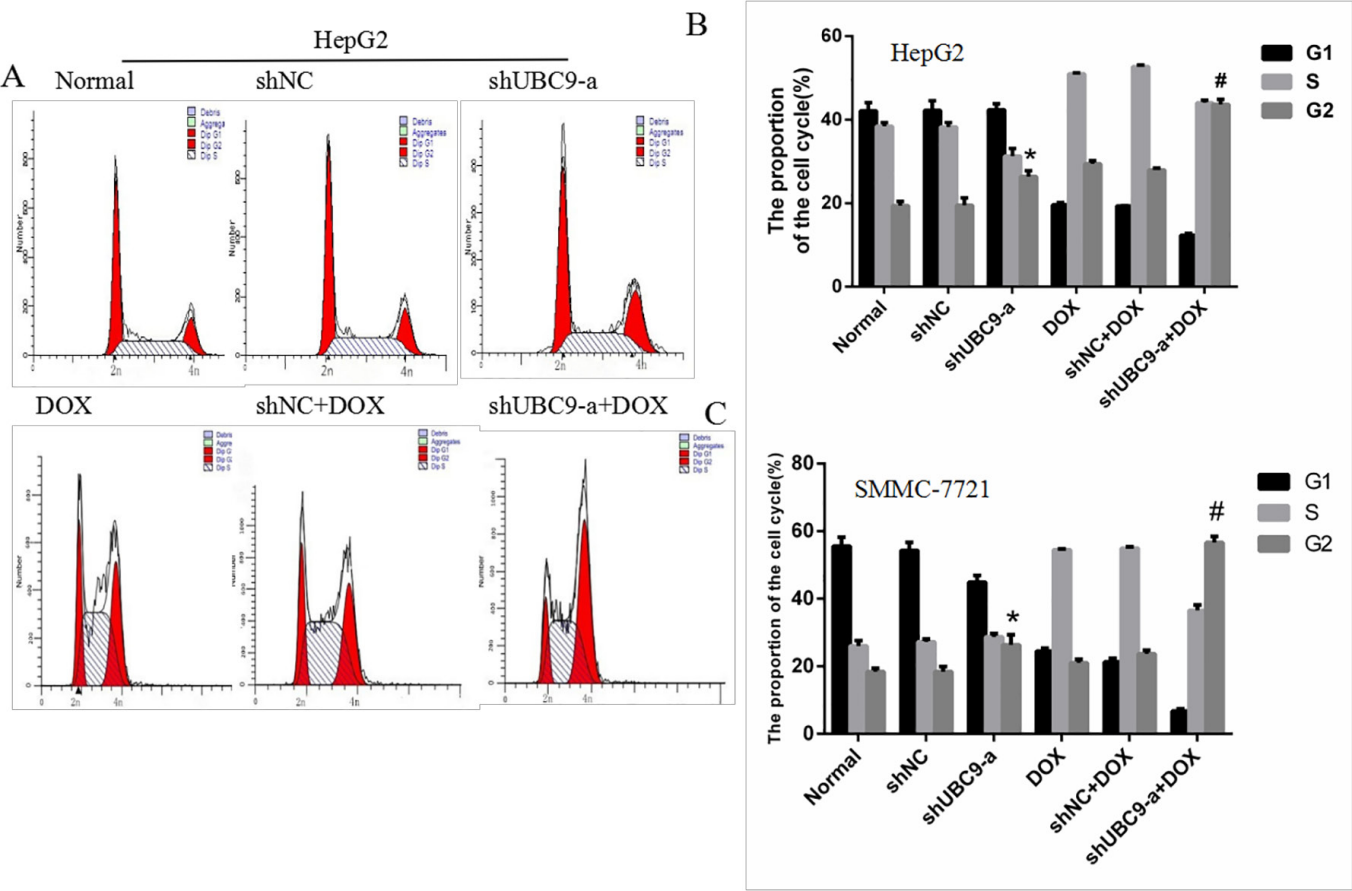
Down-regulation of UBC9 improved the DOX-induced cell cycle arrest of HCC cells (**A**) Cell cycle distribution was assessed with the Cell Cycle Analysis Kit. (**B** and **C**) HepG2 and SMMC-7721cells with UBC9 shRNA in combination with DOX showed a significantly increased rate in the G2 phase compared to cells in the Normal or NC shRNA Combination with DOX groups (^#^*P* < 0.001). The shUBC9-a group also showed a significantly increased rate in the G2 phase compared to the Normal or NC shRNA groups (*P < 0.001).

To examine whether UBC9 shRNA combined with DOX was related to the cell cycle, we examined cell cycle progression using flow cytometry. We used three groups of cells alone or cells treated with doxorubicin for 48 h. The cell cycle assay revealed that the shUBC9-a group had a higher number of cells in the G2/M phase compared to the Normal and shNC groups (*p* < 0.01) (Figure 6A–6C). Moreover, the cells shUBC9-a combined with DOX treatment caused more accumulation of cells in the G2/M phase compared to the other groups (*P* < 0.001). The increase in the G2/M phase cell population was accompanied by a reduction in the number of cells in the G0/G1 and S phases of the cell cycle. Therefore, UBC9 shRNA combined with DOX induced cell cycle arrest at the G2/M phase.

### The effects on the MAPK signal pathway after UBC9 shRNA was combined with DOX treatment in HCC cells

**Figure 7:**
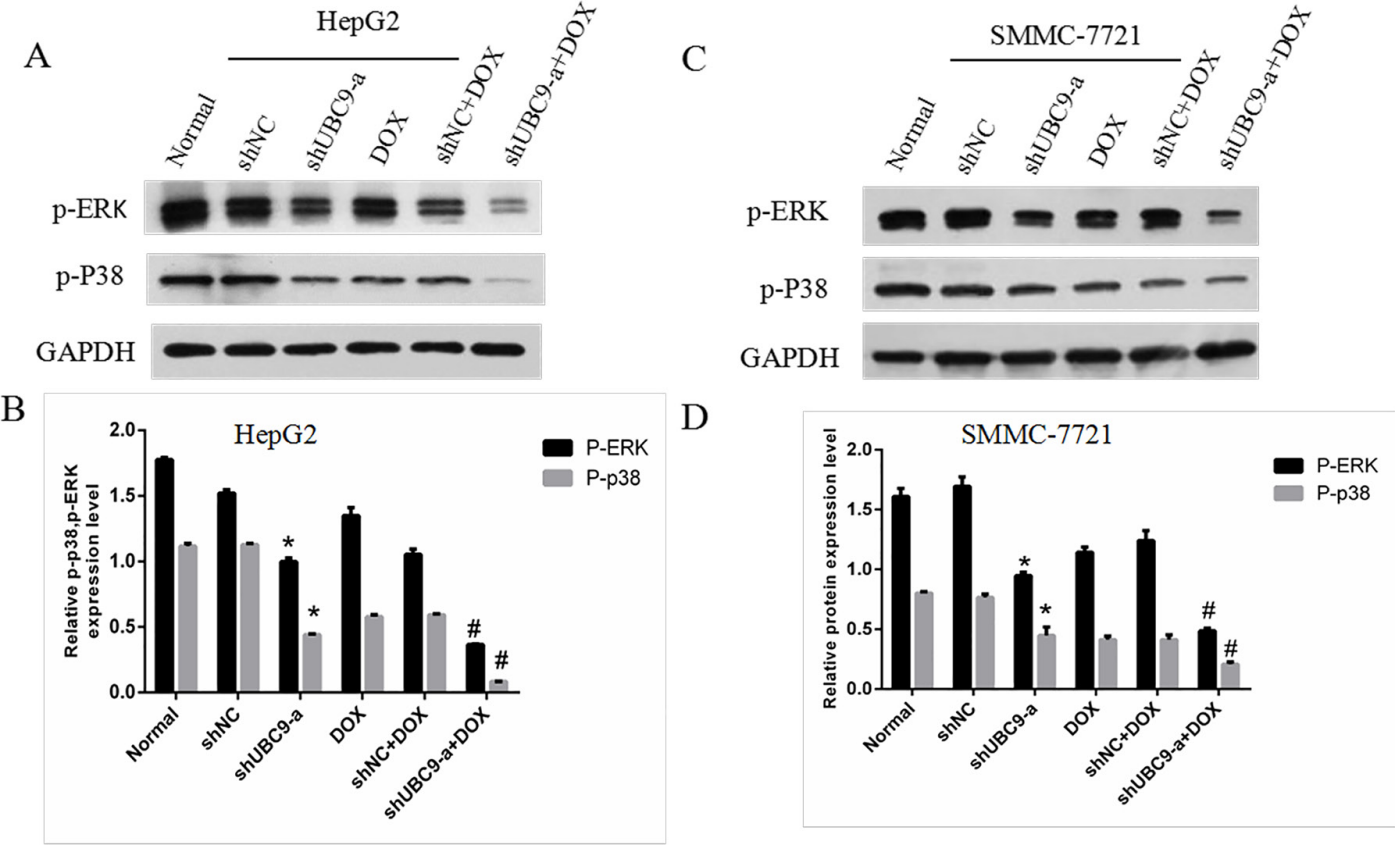
UBC9 knockdown influenced the expression and activation of multiple downstream genes (**A** and **C**) The expression of p-ERK and p-p38was evaluated by Western blot with GAPDH as a loading control. (**B** and **D**) Graph of the relative ratios of p-ERK and p-P38 protein to GAPDH in each group. *P < 0.01 vs NC shRNA transfected cells, ^#^*P* < 0.001 vs the other groups, *n* = 6.

Cell apoptosis is tightly controlled by a complex regulatory network. The MAPK pathway is required for apoptosis and can be induced by chemotherapeutic agents, and when UBC9 shRNA was combined with DOX, the MAPK pathways may play a key role in the induction of apoptosis. The expression of the p-p38 and p-ERK1/2 proteins obviously decreased in the shUBC9-a with DOX and shUBC9-a groups (*P* < 0.001 all) in HepG2 and SMMC-7721 cells, and the shUBC9-a with DOX group had a larger reduction compared to shUBC9-a alone (*P* < 0.001) (Figure 7A, 7B).

## DISCUSSION

Protein SUMOylation has been implicated in the regulation of protein stability, subcellular localization, and activity of transcription factors [35–37]. There is also evidence that suggests that SUMOylation alterations are associated with multidrug resistance in hepatocellular carcinoma [38]. UBC9 is the sole E2-conjugating enzyme required for SUMOylation. Therefore, inhibiting the expression of UBC9 could damage cell SUMOylation. There is increasing evidence that UBC9 plays an important role in human neoplasia [39–41] and in chemotherapy resistance, which has been demonstrated both in non-small-cell lung cancer and melanomas [42–43]. Recently, another study has indicated that high UBC9 expression correlates with a poor response to chemotherapy and poor clinical prognosis in breast cancer [44]. Given these findings, we know that UBC9 may have a global function in tumorigenesis and drug responsiveness.

In the present study, we investigated the effects of the down-regulation of UBC9 on chemosensitivity to doxorubicin in HCC cells and explored the underlying mechanisms. First, our data indicated that UBC9 was upregulated in HCC tissues and cells. UBC9 overexpression correlated closely with tumor size, tumor microsatellite formation, and tumor encapsulation. Second, down-regulation of UBC9 increased DOX-induced apoptosis and caused cell-cycle arrest in HCC cells. Third, inhibition of UBC9 significantly lowered the IC50 values of DOX for HCC cells, which indicated that down-regulation of UBC9 increased the sensitivity of HCC cells to DOX. Fourth, down-regulation of UBC9 resulted in a decrease in the expression of the anti-apoptotic proteins Bcl-2 and Bcl-xl as well as upregulation of the expression of the pro-apoptotic proteins cleaved-Caspase3 in HCC cells, which are important regulators of apoptosis and drug resistance [45]. Finally, the results indicate that knockdown of UBC9 may have an effect on the MAPK signaling pathway, which has an important role in both cell apoptosis and tumor cell drug resistance [46].

The extrinsic death receptor and intrinsic mitochondrial pathway can both induce death in cancer cells and sensitize them to established cytotoxic agents and radiation therapy [47]. In the present study, down-regulation of UBC9 significantly suppressed expression of Bcl-2 and Bcl-xl in addition to promoting expression of cleaved-Caspase3 combined with DOX. These data suggest that UBC9 modulates DOX-induced apoptosis via the intrinsic apoptosis pathway. Recently, drug resistance has been attributed to abrogation of the intrinsic apoptosis pathway [47]. Bcl-2, one of the most important regulators of this pathway is part of the Bcl-2 family of proteins, and upregulation of Bcl-2 expression increases resistance to chemotherapeutic drugs, whereas downregulation of Bcl-2 expression promotes an apoptotic response to anticancer drugs [45]. Additionally, chemotherapy drugs, such as doxorubicin, can lead to unnecessary cell death, whereas Bcl-xl showed a stronger protective effect than Bcl-2 against cell death induced by several chemotherapy agents [48]. In particular, Caspase3 is one of the key components of apoptosis and is responsible for the Bcl-2and Bcl-xl proteins [49]. Thus, the findings suggest that down-regulation of UBC9 significantly increased the sensitivity to DOX and that sensitivity might be partly activated by the intrinsic apoptotic pathway in HCC cells.

It was reported that SUMOylation plays an important role in MAPK signal pathway conditioning [50–52]. MAPK is an important component of cell signaling pathways and regulates many protein functions in human cells [53]. The MAPK pathways respond to various extracellular stimuli and control a large number of fundamental cellular processes, including growth, proliferation, differentiation, survival and apoptosis [54]. Mitogen-activated protein kinase (MAPK) play an important role in both cell apoptosis and tumor cell drug resistance [45]. MAPKs include extracellular signal-regulated kinase (ERK), p38, and c-Jun NH2-terminal kinase (JNK) [55]. The ERK-MAPK signaling pathways are closely related to cell drug susceptibility [56], and there is also a recognized connection between the MAPK and intrinsic apoptosis pathways. Activation of ERK1/2 inhibits BIM-induced apoptosis by interfering with the binding of BIM to MCL-1 and BCL-XL [57]. The P38-MAPK signaling pathways are closely related to stress-induced apoptosis and cell-drug susceptibility [58]. Our study showed that down-regulation of UBC9 combined with DOX inhibited ERK1/2 and P-p38 activation in HepG2 and SMMC-7721 cells. The levels of phosphorylation of ERK and P38 expression decreased in HCC, which indicated that inhibition of UBC9 increased the sensitivity of chemotherapeutic drugs through inhibition of the ERK1/2-MAPK and P38-MAPK cell pathways. These findings suggested that the increased sensitivity of HCC cells to doxorubicin induced by down-regulation of UBC9 was achieved by inhibiting the MAPK signaling pathway. The results further showed that inhibition of UBC9 gene expression could increase the sensitivity of HCC cells to doxorubicin.

In summary, we demonstrated that UBC9 was overexpressed in HCC and that its expression was correlated with clinical outcomes. Our results also showed that down-regulation of UBC9 increased the sensitivity to DOX in hepatocellular carcinoma cells. This phenomenon occurs due to decreased expression of Bcl-2 and Bcl-xl and increased expression of cleaved-Caspase3, which resulted in DOX-induced apoptosis. Furthermore, inhibition of the ERK1/2 and P38 MAPK pathways by down-regulation of UBC9 could increase the response to DOX in HCC cells. These results suggest that UBC9 may be a potential candidate for a chemosensitizer in the treatment of HCC and imply that UBC9 down-regulation might serve as a therapeutic target for hepatocellular carcinoma.

## MATERIALS AND METHODS

### Samples

Human HCC specimens were collected from 103 patients who received an HCC resection at the Second Affiliated Hospital of Nanchang University between January 2014 and December 2015. Informed consent was obtained from each patient, and the study protocol was approved by the Ethics Committee of the Second Affiliated Hospital of Nanchang University.

### Materials

HepG2 and SMMC-7721 cells were purchased from the Cell Bank of the Chinese Academy of Sciences (Shanghai, China). Lipofectamine 2000 Reagent was purchased from Transgen Biotech (Beijing, China). DOX was purchased from Nanjing Keygen Biotech. Anti-Bcl-2, anti-Bax, cleaved-Caspase 3, anti-Caspase 3 anti-GAPDH were obtained from Proteintech (Wuhan, China). Anti-p38, anti-p-p38, anti-ERK1/2 and anti-p-ERK1/2 were obtained from Cell Signaling (Boston, MA, USA). Anti-UBC9, anti-Bcl-xl, anti-JNK and anti-p-JNK were obtained from Abcam (Boston, MA, USA). Cell Counting Kit-8 (CCK8) was purchased from Bytotime Company (Nantong, Jiangsu Province, China). The Annexin V/FITC kit was purchased from BD (BioSciences, USA). The Chemiluminescence (ECL) assay kit was purchased from Amersham (Arlington Heights, USA). The mouse anti-GAPDH polyclonal antibody (1:1,0000) (Proteintech, Wuhan, China), anti-rabbit (1:10,000) and anti-mouse (1:10,000) IgGs were purchased from Proteintech (Wuhan, China).

### Cell cultures and transfections

HCC cells were cultured in Dulbecco's modified Eagle's medium (DMEM, Solarbio, Shanghai, China) supplemented with penicillin (100 U/mL), streptomycin (0.1 mg/ml), and 10% fetal bovine serum (Gibco, Grand Island, NY, USA). Cells were grown at 37°C in a 95% air and 5% CO_2_ atmosphere. HepG2 cells were transfected with either plasmids expressing UBC9 shRNA or empty vector plasmids using a Lipofectamine 2000 transfection reagent according to the manufacturer's instructions. The plasmid-transfected HCC cells were cultivated at 37°C for 24 hours. The transfected cells were divided into six experimental groups: Normal, shNC, shUBC9-a, DOX, shNC+DOX, sh-UBC9-a+DOX. After 24 hours, the transfected cells were collected for analysis of mRNA and protein expression. shUBC9-a: 5′-GGAGGAAAGACCACCCATTTG-3′; shUBC9-b:5′-GCACGATGAACCTCATG AACT-3′; shUBC9-c:5′-GCAGGCCTACACGATTTTAC-3′.

### Real-time quantitative polymerase chain reaction (qRT-PCR)

Total RNA was extracted using TRIzol (Invitrogen), and cDNA synthesis was performed using the Prime-Script RT reagent Kit (Takara, Japan). Quantitative-PCR was performed using the SYBR Premix Ex Taq ™II (Takara) on an Applied Biosystems 7300 Real-Time PCR System (ABI, USA). The UBC9 primer sequence came from PrimerBank (http://pga.mgh.harvard.edu/primerbank/). Oligomer primers were designed for the following genes: UBC9 (Sense: 5-CAG GAA AGA AAG GGA CTC-3; Antisense: 5-TTC GGG TGA AAT AAT GG-3) and GAPDH: (Sense: 5-GCA TCC TGC ACC ACC AAC T-3; Antisense: 5-GCA GTG ATG GCA TGG ACT GT-3). Each reaction had a total volume of 20 μl, including 12.5 μl of SYBR Green master mix, 200 nmol of each forward and reverse primer, 1 μl of cDNA, and 7 μl of ddH_2_O. The cycling conditions included an initial denaturation step of 10 minutes at 94°C, followed by 40 cycles of 15 seconds at 95°C, one minute at 55°C and one minute at 60°C.

### Western blot

The six experimental groups were removed from the incubator. The cells were washed with ice-cold PBS twice andharvested. Then, cell lysates were prepared using RIPA buffer supplemented with protease inhibitors (100 mM Tris, pH7.4, 150 mM NaCl, 5 mM EDTA, 1% Triton X-100, 1% deoxycholate acid, 0.1% SDS, 2 mM phenylmethylsulfonylfluoride, 1 mM sodium orthovanadate, 2 mM DTT, 2 mM leupeptin, 2 mM pepstatin). The protein concentrations were determined using the BCA protein assay (Thermo Fisher Scientific, Rockford, USA). Then, 10 μl of each sample was separated by SDS–polyacrylamide gel electrophoresis and transferred to a PVDF membrane (0.22 micrometer, Bio-Rad) and then blocked with 5% skim milk at room temperature. The membrane was incubated with primary antibodies overnight at 4°C. The membrane was washed in TBST three times (10 min each time). Then, the PVDF membrane was incubated with the appropriate secondary antibody at room temperature for 2 h. The PVDF membrane was washed in TBST three times (10 min each time). The proteins were visualized using the Amersham^™^ ECL Plus Western Blotting Detection System (GE Healthcare, UK).

### Cell viability assay

To evaluate the proliferation effect of UBC9 in HCC cells, shUBC9-a and shNC stable cells were digested and counted, and 1 × 10^4^ cells in 100 μl medium were seeded in 96-well plates. After a 24 h incubation, the cultured cells were adherent. Different concentrations of DOX (0, 0.1, 0.2, 0.4, 0.5, 0.8 and 1.6 μg/ml) were added to the medium. After incubating the cells at 37°C (5% CO_2_) for 24 h, 10 μl of CCK-8 cells (Bytotime, Jiangsu, China) were added to the medium. After the cells were incubated for another 2 h at 37°C (5% CO_2_), we measured the opticaldensity (OD) at 450 nm on a microplate reader (Bio-Rad, California, USA). The relative inhibition rate = (OD of control group − OD of drug group)/OD of control group × 100%. We used SPSS 18.0 to calculate the half maximal inhibitory concentration (IC50).

### Apoptosis assay by AnnexinV-FITC/PI staining

Cells (5 × 10^5^) were seeded in 6-well plates. After the cells were transfected, 0.3 μg/ml DOX was added to the medium, and the cells were incubated for 24 h. We harvested the adherent cells, washed the cells twice with cold PBS and then resuspended the cells in 1 × Binding Buffer at a concentration of 1 × 10^6^ cells/ml. Then 100 μl of the solution was transferred to a 5 ml culture tube. Next, 5 μl of AnnexinV-PE and 5 μl of PI were added (BD BioSciences, USA). The cells were gently vortexed and incubated for 15 min at RT (25°C) in the dark. Finally, we added 400 μl of 1 × binding buffer to each tubeaccording to the manufacturer's instructions. The stained cells were examined with FACSCalibur flow cytometry.

### Cell-cycle phase distribution assay

Cells (5 × 10^5^) were seeded in 6-well plates after transfection, and then,0.3 μg/ml DOX was added to the medium. After incubation for 48 h, the adherent cells were collected and washed twice with cold PBS. Then, the cells were fixed in ice cold 70% ethanol. The cell cycle distribution was analyzed using the Cell Cycle Analysis Kit (MultiSciences, China). The percentage of cells in different phases of each cell cycle was sorted using a ModFit 5.2 computer program.

### Immunohistochemistry

The level of UBC9 in paraffin-embedded tissue sections was detected with immunohistochemical staining. A goat monoclonal antibody was used as the primary antibody. Paraffin-embedded tissues were pretreated at 65°C for 2 h, followed by deparaffinization using standard procedures. Antigen retrieval was carried out with an antigen retrieval solution (10 mmol/L Tris, 1 mmol/L EDTA, Ph 9.0) before 2% sheep serum was added. Then, the slides were incubated with the antibody for UBC9 (ab21193, Abcam) (1:200 dilution) at 4°C overnight. Next, the slides were labeled with EnVision HRP kits (DAKO) at room temperature for 30 minutes, incubated with DAB substrate liquid (DAKO), and counterstained with Mayer› s hematoxylin(DAKO). All of the sections were observed and photographed with a light microscope using a DP70 CCD system (Olympus Corp.).

### Statistics

All data were analyzed using SPSS 18.0. The results are presented as the means ± SD from three independent experiments. The differences between the groups were analyzed by the Student t test when two groups were compared or by one-way ANOVA when more than two groups were compared. The correlations between the UBC9 expression levels and clinical pathological variables were analyzed using Pearson's Chi-squared test. The test results were considered significant at *P* < 0.05.
